# Prognostic Value of Troponin I for Infarct Size to Improve Preclinical Myocardial Infarction Small Animal Models

**DOI:** 10.3389/fphys.2015.00353

**Published:** 2015-11-27

**Authors:** Aurélien Frobert, Jérémy Valentin, Jean-Luc Magnin, Erwin Riedo, Stéphane Cook, Marie-Noëlle Giraud

**Affiliations:** ^1^Cardiology, Department of Medicine, University and Hospital of FribourgFribourg, Switzerland; ^2^Central Laboratory, Hospital FribourgFribourg, Switzerland

**Keywords:** myocardial infarction, troponin I, infarct size, prognostic value

## Abstract

Coronary artery ligations to induce myocardial infarction (MI) in mice and rats are widely used in preclinical investigation. However, myocardial ischemic damage and subsequent infarct size are highly variable. The lack of standardization of the model impairs the probability of effective translation to the clinic. Cardiac Troponin I (cTnI) is a major clinically relevant biomarker.

**Aim:** In the present study, we investigated the prognostic value of cTnI for early estimation of the infarct size.

**Methods and Results:** Infarcts of different sizes were induced in mice and rats by ligation, at a random site, of the coronary artery. Kinetics of the plasma levels of cTnI were measured. Heart function was evaluated by echocardiography, the percentage of infarcted left ventricle and infarct expansion index were assessed from histological section. We observed that plasma cTnI level peaked at 24 h in the infarcted rats and between 24 and 48 h in mice. Sham operated animals had a level of cTnI below 15 ng/mL. Infarct expansion index (EI) assessed 4 weeks after ligation showed a large variation coefficient of 63 and 71% in rats and mice respectively. We showed a significative correlation between cTnI level and the EI demonstrating its predictive value for myocardial injury in small animal models.

**Conclusion:** we demonstrated the importance of cTnI plasma level as a major early marker to assist in the optimal and efficient management of MI in laboratory animals model. The presented results stress the need for comparable biomarkers in the animal model and clinical trials for improved translation.

## Introduction

Heart disease remains a major cause of mortality worldwide. New treatment strategies using gene and cell therapies have gained interest because their efficacy has now been established in a preclinical setting. However, their translation into clinical practice has been disappointing (Houser et al., 2012). Although numerous phase 1 clinical trials have demonstrated the safety and feasibility of new therapies that rely on the delivery of cells to the myocardium, the efficacy of the treatments have not been as convincing or effective as suggested in the preclinical studies. The reasons for these shortcomings are multifactorial, and general recommendations have been proposed to improve translational research in heart failure (Patten and Hall-Porter, 2009). The scientific community believes that the optimization of preclinical studies in small animal models is of utmost importance for developing safe and effective therapies and increasing the probability of effective translation to the clinic (Houser et al., 2012).

Rat and mouse models of myocardial infarction (MI) are widely recognized standard preclinical approaches used for the assessment of new potential therapies (Patten and Hall-Porter, 2009). MI resulting from a coronary thrombosis is commonly simulated by permanent coronary artery ligation in rodent and murine models. These models have been continuously improved since 1960, leading to a drastic reduction in post-operative mortality. However, one of the principal limitations of these models is the large variability in the infarct size (Degabriele et al., 2004). The size of the infarct depends on the position of the coronary ligature and coronary collateral circulation, which varies from one individual to another (Toyota, 2005). It is necessary to correctly evaluate the infarct size when assessing remodeling or repair responses to a regenerative treatment (Houser et al., 2012). The effectiveness of the model for a bias-free evaluation of novel therapies requires an estimation of the infarct size pre and post treatment.

Several techniques are currently available to gauge the myocardial infarct size; however, they are imprecise (e.g., estimating the coronary ligature position and consequent bleaching zone); demanding in terms of time, expertise, and equipment (e.g., echocardiography or magnetic resonance imaging); and only available for end-point evaluations (e.g., histological assessment of fibrosis). While quantification of myocardial injury using biomarkers is common in clinical settings, it is not used in preclinical investigations. Cardiac troponins (cTn), particularly the I and T isoforms, are well-established biomarkers for the diagnosis and prognosis of cardiac injury and play a key role in the risk stratification of acute coronary syndromes (Thygesen et al., 2010; Hallén, 2012; Hall et al., 2015). The cardiac isoforms are highly specific and sensitive for the evaluation of the extent of myocardial necrosis. In addition, cTnI release from the injured myocardium can be reliably detected early after the myocardial infarction.

The purpose of this study was to investigate the prognostic value of cTnI for the quantification of MI size in small animal models (rat and mouse) to improve the coronary artery ligation experimental model.

## Materials and methods

### Animals

All animals received care in compliance with the European Convention on Animal Care. The surgical procedures were performed in accordance with the Swiss Animal Protection Law after obtaining permission from the State Veterinary Office, Fribourg and approval from the Swiss Federal Veterinary Office, Switzerland (2013_51_FR and 2013_09_FR). All surgical interventions were performed under isoflurane anesthesia. Effort was made to diminish any animal suffering; specifically, all animals received a subcutaneous injection of 0.1 mg·kg^−1^ Temgesic (buprenorphine, 0.3 mg·ml^−1^; ESSEX Chemie; Luzern, Switzerland) after surgery.

### Surgery

In total, 28 female Lewis rats (218 ± 9 g) and 23 NMRI female mice (49 ± 5 g) (Janvier; Le Genest, France) were included in this study.

As previously described (Frobert et al., 2014), the animals were anesthetized with isoflurane and oxygen (5% for induction and 2.5% for maintenance). The animals were placed on a warming pad at 37°C to avoid hypothermia during anesthesia and ventilated with a 14-G IV cannula for the rats and 20-G IV cannula for the mice (Abbocath, Abbott; Sligo, Ireland) at 80 cycles per minute (adapted to weight; Harvard Inspira Apparatus, Inc.; Holliston, MA, USA). The hearts were accessed through a left thoracotomy between the fourth and fifth interstitial space. After opening the pericardium, a permanent ligation of the left anterior descending coronary artery (LAD) was performed (7/0 polypropylene suture, Ethicon, Inc.; Somerville, MA, USA). Three mice and three rats were sham operated without a coronary ligation. Three healthy mice and three healthy rats that did not undergo surgery were also included as controls.

### Blood sampling and cTnI assay

Blood was collected in Litium/Heparin tubes from the caudal tail artery and jugular vein in the anesthetized rats and mice, respectively, at various time points (2, 5, 24, 48, and 72 h) after the LAD ligation. The blood was centrifuged, and the plasma was immediately stored a −80°C for up to 2 weeks. Plasma was diluted 1/5 for rats and 1/7 for mice in AccuTNI3+ buffer (Beckman Coulter, Switzerland). cTnI was quantified with the AccuTNI3+ immunoassay using a UniCel DxI 800 system (Beckman Coulter, Switzerland). The immunoassay detects human cTnI with an analytical measuring range of 0.01–80 ng/mL. The immunoassay also cross-reacts with mouse and rat cTnI (Apple et al., 2008). The assays were performed initially in triplicate for validation and then only once.

### Echocardiography

Echocardiograms were recorded 2 and 6 weeks after LAD ligation in a blinded manner by an experienced cardiologist as previously described (Guex et al., 2014). Briefly, animals were anesthetized with 2.5% isoflurane and placed in a left lateral position. Images were recorded using a 15 MHz linear array transducer system (Acuson, Sequoia C512, Siemens, Inc.; Malvern, PA, USA). The fractional shortening (FS) and ejection fraction (EF) were estimated based on the M-mode or a two-dimensional analysis, respectively.

### Histopathological sampling and analysis

The hearts were harvested, washed in KCl (1 M), and stored at −20°C for 1 h in an acrylic heart matrix (Harward apparatus). The hearts were cut with a razor blade into 2- or 1-mm sections from the rats and mice, respectively: 6–8 sections were obtained from the base to the apex. Each section was embedded in paraffin using a standard histological procedure. The paraffin blocks were sectioned at 5-μm intervals with a rotary microtome and stained with Masson-Goldner trichrome staining. The slices were successively incubated in Mayer's Hematoxylin (Merck AG; Zug, Switzerland), Acid Fuchsin-Ponceau (Sigma-Aldrich; Buchs, Switzerland), Phosphomolybdic Acid Orange G (Merck AG; Zug Switzerland and Sigma-Aldrich; Buchs, Switzerland), and Lichtgrün (Sigma-Aldrich; Buchs, Switzerland) solutions. The samples were dehydrated with an ascending ethanol series and mounted with Eukitt (EM Sciences; Hatfield, PA, USA). Images were acquired with a stereomicroscope Nikon SM2 800 mounted with a Nikon 1 camera (Nikon; Tokyo, Japan).

Bersoft Image Analysis software (Bersoft Technology and Software; Lunenburg, Canada) was used to measure the scar thickness in the middle of the infarct, septum thickness, left ventricle (LV) cavity area, infarct area, and LV tissue area. The infarct expansion index (EI) was calculated as ([LV cavity area/whole LV area]/[infarct thickness/septum thickness]); the whole LV area was measured as the combined LV cavity and LV tissue area (Landa et al., 2008). The size of the infarct was calculated as the percentage of the LV area (infarct area/LV tissue area). The measurements were performed on one slice of each heart section and averaged according to Takagawa et al. (2007).

### Statistics

The results are presented as mean ± standard deviation. Linear regression analyses were performed using Prism software. The non-parametric Spearman test was computed in a two-tailed manner. The non-parametric Wilcoxon matched pairs test was used to compare two paired groups. Values were considered significantly different when *p* < 0.05. Variability was estimated using coefficients of variance (%CV).

## Results

### Myocardial infarction

A total of 28 rats and 23 mice were included in the study. The MI group consisted of 22 rats and 16 mice that underwent a LAD ligation. Peri-operative mortalities were low (no rat, one mice). Each of the sham and healthy groups comprised three rats and three mice.

**Figure 1:**
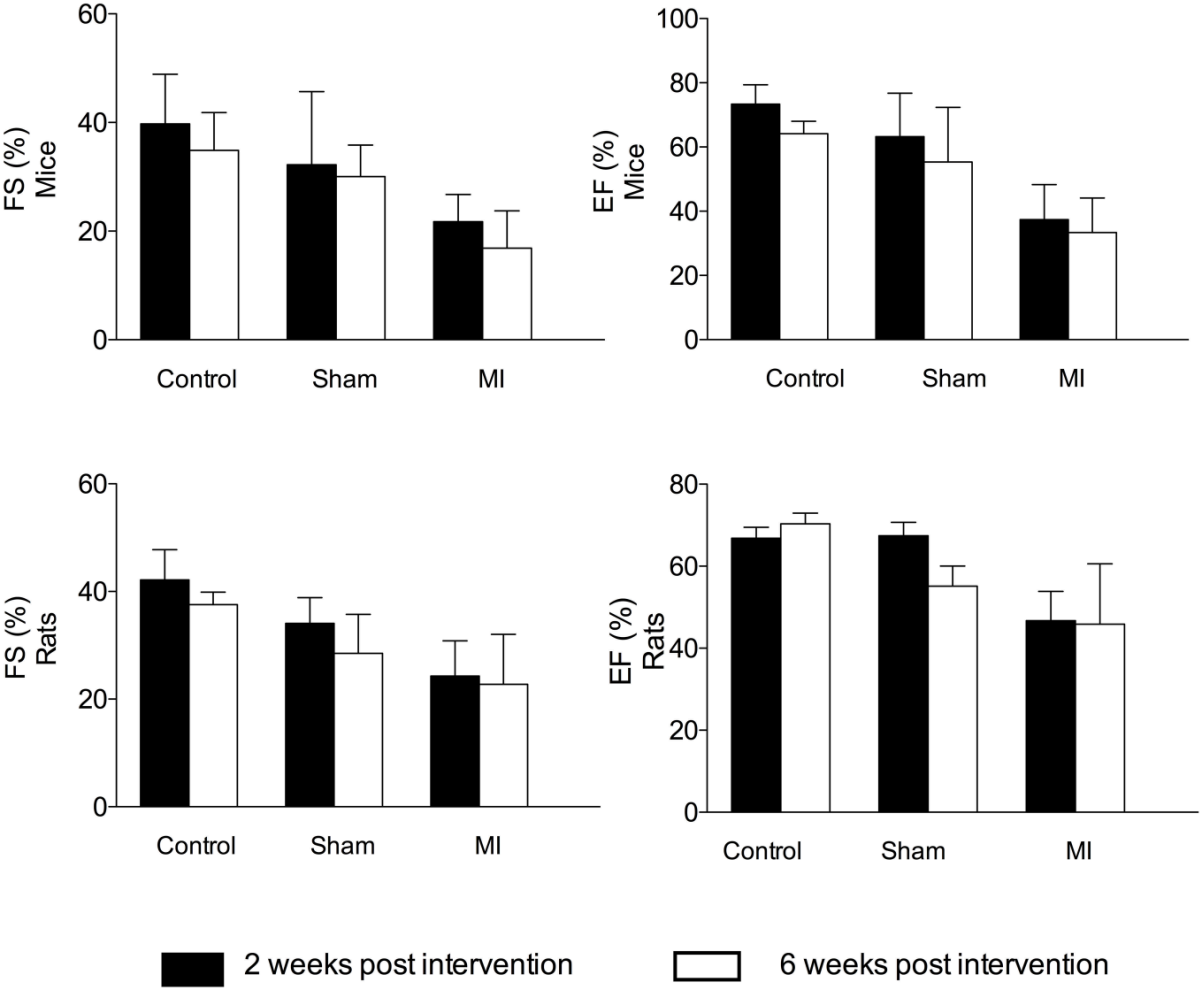
**Longitudinal echocardiographic assessments of heart function performed at 2 weeks (black bars) and 6 weeks (white bars) after the time of surgery are represented for mice (above) and rats (below)**. The fractional shortening (FS) was calculated using the M-mode and ejection fraction (EF) from the two-dimensional imaging mode. The animals were healthy (control group, *n* = 3 mice and rats), sham-operated (*n* = 3 mice and rats), or received a LAD ligation (*n* = 8 mice and *n* = 15 rats).

The LAD ligations resulted in a reduction in heart function, and there was a significant difference between the LAD-ligated groups and the healthy and sham groups. The FS and EF are presented in Figure 1. The ligations were performed at a random site on the coronary artery. Accordingly, an assessment of the heart function showed large variability on the echocardiogram at the 2-week follow-up. For the mice, the EF ranged from 23–59% (mean EF = 37 ± 11, CV = 29%). The sham-operated and healthy animals had higher EF values that varied from 50–77% (mean EF = 63 ± 14, CV = 21%) and from 66–77% (mean EF = 73 ± 6, CV = 8%), respectively.

At 2 weeks post-ligation, the minimum and maximum EF values for the rats were 34% and 56%, respectively (mean EF = 44 ± 6, CV = 13%). For the sham and healthy groups, the minimum and maximum EF values were 64 and 70% (mean EF = 67 ± 3, CV = 5%) and 64 and 70% (mean EF = 67 ± 3, CV = 4%), respectively.

After 6 weeks, the average EF and FS remained stable in both the mice and rats in all of the groups compared with the measurements performed at 2 weeks post-ligation. No significant differences in FS and EF were observed between 2 and 6 weeks post LAD ligation (Figure 1).

**Figure 2:**
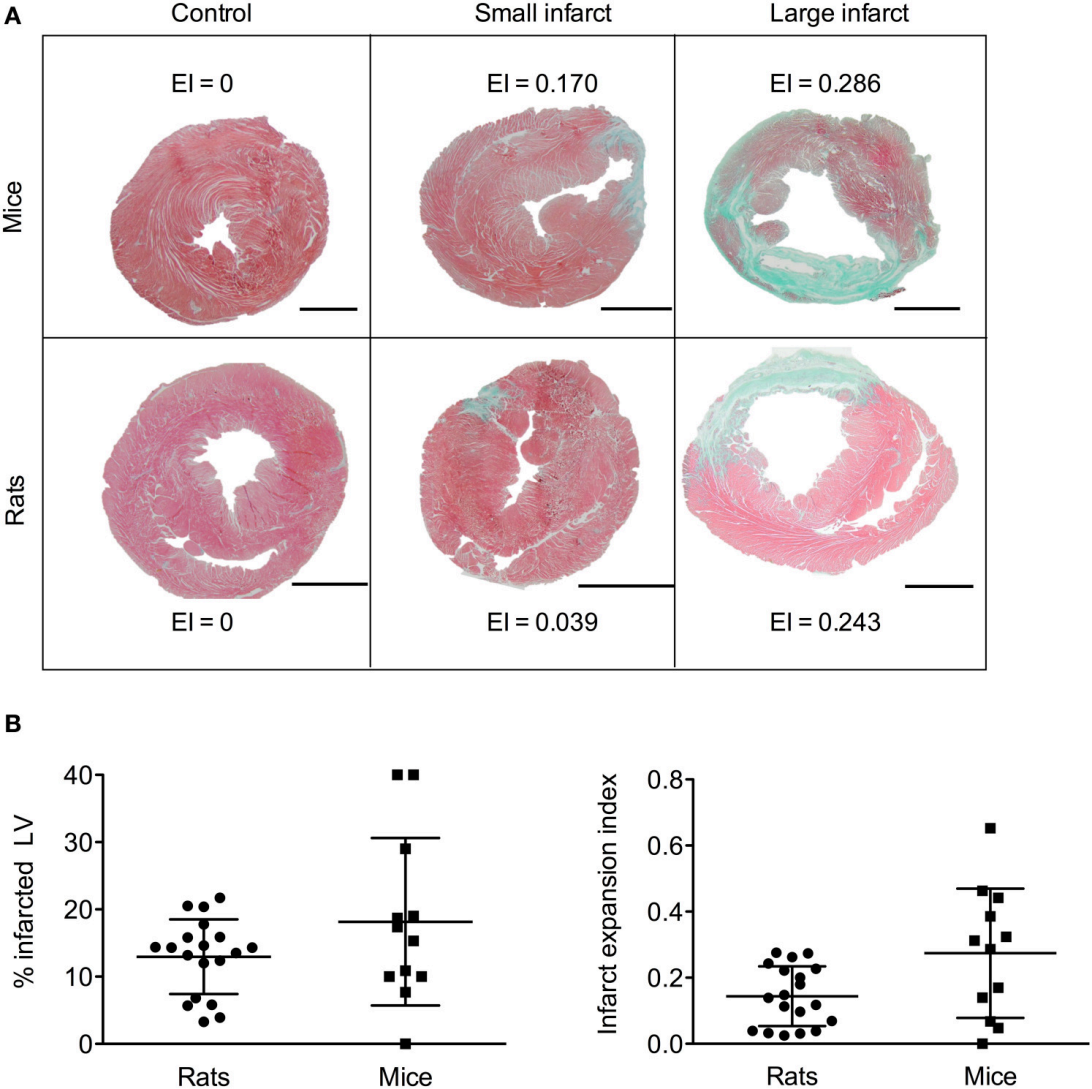
**(A)** Representative sections with Masson-Goldner trichome staining showing healthy myocardia in red and fibrotic tissues in green. Scale bars indicate 1.5 mm for mice and 3 mm for rats. The sections were from a healthy control and LAD ligated animal, respectively. **(B)** Myocardial injury was assessed as the percentage of the infarcted area of the left ventricle and the infarct expansion index in mice and rats at 6 weeks post LAD ligation.

The Masson-Goldner trichrome stainings were performed 6 weeks after the LAD ligation to assess the infarct size and expressed as a percentage of the LV and the EI. As illustrated in Figure 2A, the histological measurements showed a wide panel of myocardial injury, with large to small infarcts within the MI group. The lesions increased from base to apex. The sham group did not display any infarcts.

In ligated rats, the EI varied from of 0.025 to 0.276 and revealed a wide range of myocardial infarct /CV of 63%; Figure 2B). The dynamic range of the % of LV was low with a minimum of 3.3% and a maximum of 21.7% (CV of 43%). The dynamic range of EI was broader (CV of 63%). When expressed as a percentage of the LV, the estimation of the infarct size may be biased. Indeed, thinning of the LV wall from remodeling-induced transmural infarct is not included in the calculation. The EI showed higher accuracy for infarct size variability estimation.

In mice, the dynamic ranges of EI and %LV were similar with respective CV of 71 and 68%.

### Kinetics of the appearance of cTnI in the blood plasma following MI

**Figure 3:**
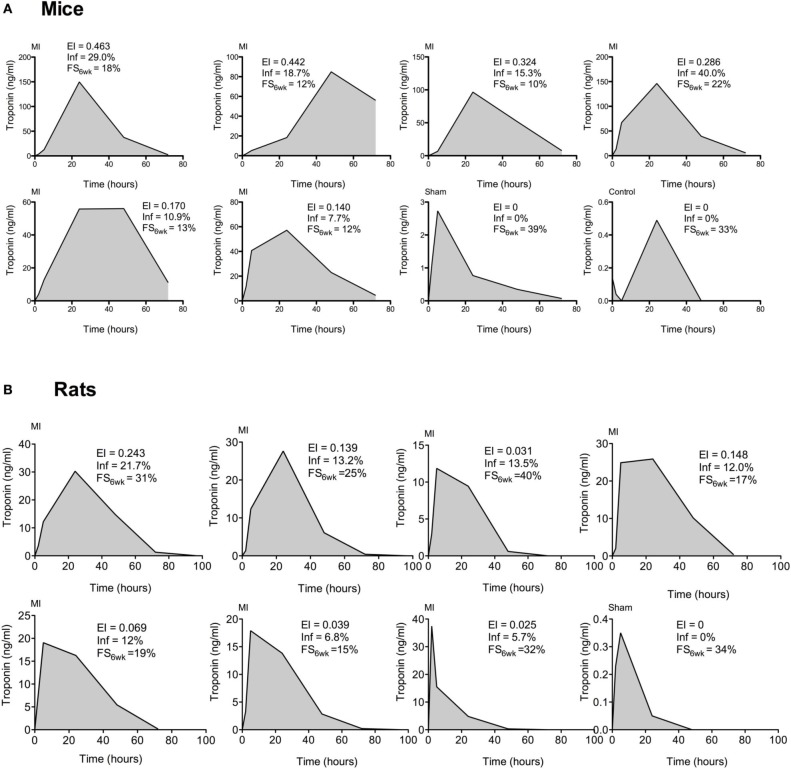
**Representative individual kinetics of plasma cTnI for mice (A) and rats (B): cTnI levels were measured at 2, 5, 24, 48, and 72 h post LAD ligation (MI) or in sham-operated animals (sham)**. Y-axis scales have been individually optimized for each graph. The peak and the area under the plasma drug concentration-time curve (AUC) are proportional to the myocardial damage, which are expressed as the expansion index (EI) and % of infarcted left ventricle (Inf). The FS recorded 6 weeks post-MI is also reported.

The individual kinetics of the plasma concentration of murine and rodent cTnI are represented in Figures 3A,B, respectively. The cTnI concentration peaked between 24 and 48 h in the infarcted mice, and it varied between the individual animals. The CV of the area under the curve (AUC) was 28%. For the sham animals, the cTnI levels were either below the detection limit or lower than 15 ng/mL.

Following LAD ligation, the plasma level of cTnI in the rats peaked at 24 h for the large infarcts (Figure 3). An earlier elevation of plasma cTnI was also observed in animals with the lowest myocardial damage (EI < 0.07). The CV of the AUC was 42%. The sham-operated animals had a level of cTnI below 3 ng/mL at 24 h.

### Correlation between cTnI and infarct size

**Figure 4:**
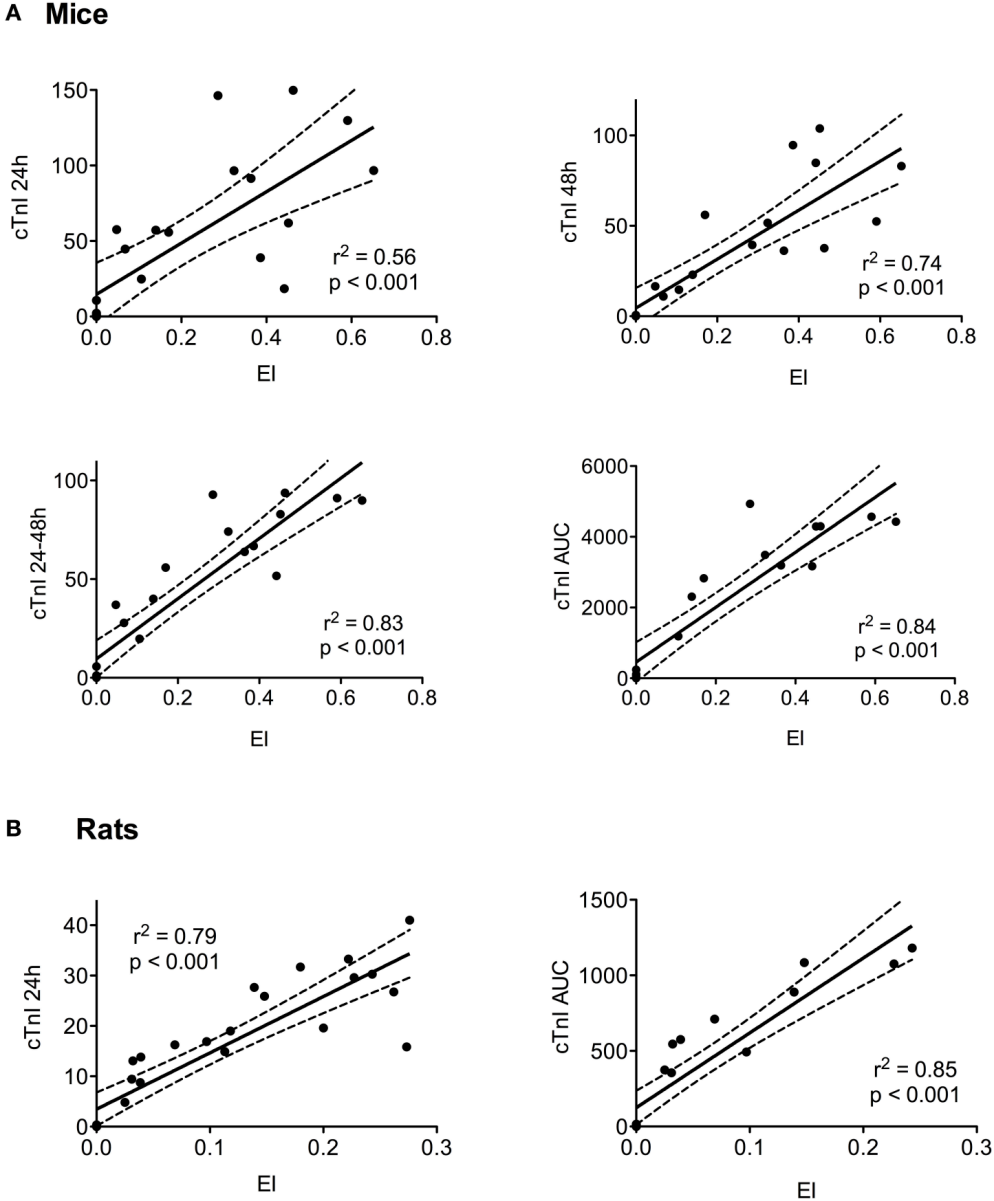
**Relationship between the expansion index (EI) and cTnI level: the EI was measured in all animals, including the healthy controls, sham-operated, and infarcted animals, 6 weeks after surgery in the mice (A) and rats (B)**. The cTnI concentrations are presented at 24 and 48 h, as the mean of the 24–48 h time period, and as the AUC. The *r*^2^ and *p*-values from the regression and correlation analyses are shown for each graph. The linear regression (black line) and 95% confidence intervals (dotted lines) are represented.

The correlations between the level of cTnI and myocardial injury measured 6 weeks after LAD ligation are presented in Figure 4. For mice, we observed significant correlations between the EI and plasma troponin levels at 24 and 48 h, mean value between 24 and 48 h, and AUC. The highest *r*^2^ were obtained between the EI and the mean 24–48 h value (*r*^2^ = 0.83, *p* < 0.001) or the cTnI AUC (*r*^2^ = 0.84, *p* < 0.001). For rats, significant correlations were obtained between the EI and level of cTnI measured at 24 h and the AUC, *r*^2^ of 0.79 and 0.85, respectively.

### Correlation between cTnI and heart function

**Figure 5:**
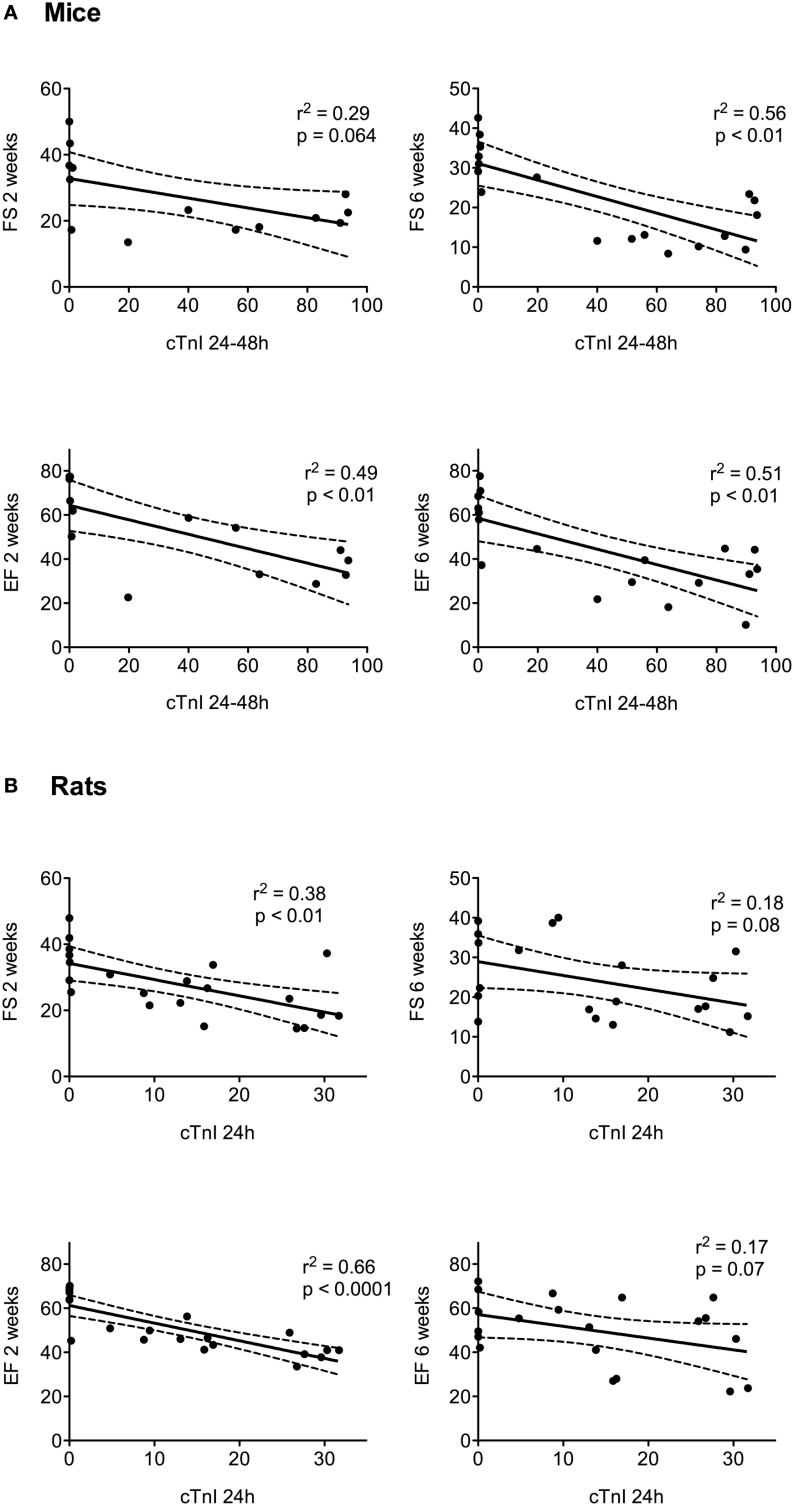
**The relationship between heart function (FS and EF) at 2 and 6 weeks after surgery and cTnI levels are represented as the mean value at 24–48 h for mice (A) and the level at 24 h for rats (B)**. The *r*^2^ and *p*-values from the regression and correlation analyses are shown for each graph. The linear regression (black line) and 95% confidence intervals (dotted lines) are represented.

The correlation between the cTnI levels and heart function, represented by the FS and EF assessed at 2 and 6 weeks after LAD ligation with echocardiography, were analyzed (Figure 5). For mice, the selected value for the cTnI level was the mean of the 24 and 48 h plasma concentrations; the peak level at 24 h was used for rats. As shown in Figure 5A, in mice, a significant correlation was found between cTnI and heart function, with a maximum *r*^2^ of 0.56. In rats (Figure 5B), we observed a significant correlation between the EF, FS, and cTnI level only at 2 weeks post LAD ligation (maximum *r*^2^ was 0.66).

## Discussion

Monitoring troponin levels in the body is an important tool for clinicians (Babuin and Jaffe, 2005; Thygesen et al., 2010). However, the use of cardiac biomarkers in preclinical models is almost only used in toxicology following administration of cardiotoxic drugs (O'Brien et al., 2006; Engle et al., 2009; Clements et al., 2010); it is rarely employed in LAD ligation MI models (Borst et al., 2011; Gomes et al., 2013).

Discrepancies have been found between preclinical and clinical studies of MI, which have emphasized the need for improved small animal models. A key limitation of the LAD ligation model is the intrinsic variability of MI size. Degabriele et al. (2004) showed that the infarct size varied from 8–65% depending on the ligature site. To assess the therapeutic efficacy of a treatment, preclinical investigations often use MI size as an end-point outcome without evaluating the infarct size before treatment or using a biomarker-guided prediction of myocardial injury. Therefore, the resulting high inter-animal variability impairs the comparison between the treated and non-treated groups and the accuracy of the therapeutic efficacy.

In this study, we demonstrated that the plasma level of cTnI can be easily quantified in a sensitive manner in mice and rats. We confirmed that a rapid rise in cTnI plasma levels occurred following LAD ligation. Furthermore, we validated the use of plasma cTnI as an early maker of myocardial injury and, importantly, provided evidence of its predictive value for myocardial injury in small animal models.

Numerous studies have validated the plasma level of cTnI in animals using a model of cardiac toxicity induced by isoproterenol (O'Brien et al., 2006; Apple et al., 2008; Schultze et al., 2011). Indeed, it has been shown that commercially available immunoassays for human cTn provide sensitive and reproducible measurements of cTn levels in various laboratory animals, including rats and mice (O'Brien et al., 2006). Using the AccuTNI3+ Beckmann immunoassay, our findings corroborate this previous study. When cTnI concentration were measured on the same samples, the chemiluminescence-based immunoassay, AccuTNI3+, showed a higher sensitivity compared to commercially available mice specific ELISA immunoassays (data not shown). Absolute values are specific to the assay used. Nevertheless, the kinetics of cTnI appearance and clearance were similar with all assays.

The detection of cTnI required either one measurement at 24 h for rats and two measurements at 24 and 48 h for mice, making it appropriate for small animal models. Importantly, we did not observe any cTnI in animals that received a sham operation, suggesting that general muscle injury from surgery does not interfere with cTnI detection.

We obtained a wide representation of infarct sizes by varying the sites of ligation. The EI and percentage of infarcted LV varied accordingly. In agreement with a study from Takagawa et al. (2007), we observed that the EI is more accurate for evaluation of the severity of myocardial damage than the percentage of infarcted LV. Indeed, the EI calculation includes more measured parameters, such as infarct thickness relative to septum thickness. This parameter is critical for chronic models of MI that present large inter-individual variability in remodeling and transmural MI such as the rat LAD ligation model. By contrast, the mouse model appeared less prone to inter-individual variability in remodeling and wall thinning.

We found that the cTnI plasma levels were strongly correlated with the EI when assessed at 2 and 6 weeks after LAD ligation. We also discovered strong correlations between the EI and the peak troponin I level and AUC values. cTnI levels with the highest *r*^2^ were selected as the optimal time point for blood sampling: 24 h post LAD for rats and 24 and 48 h for mice (the mean value had the highest correlation with the EI).

Previously, in acute MI, Vietta et al. (2013) reported that the cTnI plasma concentration assessed 8 h after injury was predictive of the myocardial damage found after 14 days in rats. Additionally, the cTnT level assessed at 24 h after LAD ligation has also been correlated with the size of the infarct in mice (Metzler et al., 2002). In addition, the plasma concentration of cTnI (measured 1 day post MI) as marker of infarct size has been recently used in mice and validated by the classic 2-3-5-triphenyltetrazolium-chloride assay performed 2 days post MI (Santulli et al., 2015).

In contrast to acute MI, chronic MI may result in remodeling of the LV. Indeed, to the best of our knowledge, this study provides the first evidence that the cTnI level can predict the size of a 6-week-old infarct in both mice and rats.

Taken together, our results indicate that the cTnI plasma level can predict MI development within the first 24–48 h. The data further suggest that the plasma level of cTnI can be used as an early inclusion/exclusion criterion for selection in preclinical experiments. This will improve group homogeneity, reducing the number of animals needed, and further enhance the quality of the experimental model. In addition, our results illustrate that the systematic use of cTnI can provide valuable predictive information for infarct size. One can envision a cardiac troponin-guided approach for evaluation of regenerative therapies for infarct size reduction.

cTnI is a widely recognized, highly specific and sensitive biomarker for myocardial necrosis. Ingkanisorn et al. (2004) demonstrated that the peak troponin I level correlated with the infarct size measured by magnetic resonance imaging in patients with ST-elevation MI. A recent large clinical study observed that cTnI measured from 16 to 24 h following primary percutaneous coronary intervention strongly correlated with the infarct size and clinical outcomes throughout a 3-month follow-up after an acute percutaneous intervention (Hall et al., 2015). Furthermore, Hallén (2012) performed a review of clinical trials and found that the coefficients of correlation between cTnI and infarct size varied between 0.53 and 0.76 and that the correlation improved with the development of more sensitive assays. Our results in these animal models suggest that cardiac troponin could also be employed as a surrogate test for infarct size prediction in trials to monitor the patient's response to treatment. Nevertheless, a full time course of cTnI plasma level may not always be available in patients and the time of the myocardial infarct may not be always identified. In addition, further investigations are necessary to define the mechanism of cTnI release, the configuration of the protein in the plasma (native or degraded protein or associated with other molecules) and its related solubility.

Echocardiographic evaluation of cardiac function and remodeling is often used as a selection criterion for MI. This technique remains a reliable, readily available, affordable, non-invasive assessment of cardiac function in small animals (Ram et al., 2011). However, clinical equipment is most commonly used for echocardiography in small animal models. In the present study, a longitudinal assessment of cardiac function was performed with a transthoracic echocardiogram using a clinical 15 MHz probe and performed by an expert cardiologist to reduce inter-observer variability. Light anesthesia, heart rate, and body temperature were maintained to ensure reliability of the measurements. B-mode and M-mode imaging were acquired at 2 and 6 weeks after LAD ligation, which allowed assessments of the EF and FS. The decrease in heart function we observed confirmed the development and progression of MI in these animal models, in agreement with previous reports (Patten and Hall-Porter, 2009). There were significant correlations between the cTnI levels and heart function when assessed 2 weeks post LAD ligation. Six weeks after the induced infarction, significant correlations were observed for mice but not for rats.

Emerging high-resolution ultrasounds have been shown to be a powerful tool for the evaluation of cardiac function (Foster et al., 2011) and should greatly improve the accuracy of global and regional cardiac function assessment, as well as remodeling, in mice. Therefore, further investigations should be undertaken to evaluate whether the weak *r*^2^ we observed may be enhanced when better heart function measurements are made using high-resolution ultrasound.

In conclusion, taken together, our results indicate that the plasma cTnI level can predict infarct expansion better than conventional echocardiographic assessments and suggest that cTnI is a robust biomarker for MI development in small animal models. This provides further prognostic information for cardiac echocardiography. Using a prognostic biomarker such as TnI also helps to define animal pre-selection for standardization of the group and may lead to a more appropriate interpretation of outcomes following experimental therapy. The use of biomarkers will improve experimental investigations to assess the effects of new therapeutics interventions.

## Author contributions

AF performed the animal experimentation; JV performed the histology; JM performed the assays optimization; ER performed the biochemical analyses; SC performed the echocardiography analyses; and MG supervised the project and wrote the article.

## Funding

The study was supported by the Swiss national foundation [SNF 310030-149986 to MG], the University of Fribourg and the Fribourg Hospital.

### Conflict of interest statement

The authors declare that the research was conducted in the absence of any commercial or financial relationships that could be construed as a potential conflict of interest.
